# Awareness During Anaesthesia

**Published:** 2009-04

**Authors:** K Sandhu, HH Dash

**Affiliations:** 1 Senior Consultant, Max Institute of Neurosciences, Saket, New Delhi; 2 Chief of Neurosciences Center & Prof. and Haad Neuroanaesthesiology, AIIMS, New Delhi

**Keywords:** Intraoperative, Awareness, Anaesthesia

## Abstract

**Summary:**

Awareness is the postoperative recall of sensory perception during general anaesthesia. The incidence is quoted at 1-2 per every 1000 patients. This rare but serious adverse event can be extremely distressing for both the patient as well as the anaesthesiologist. Awareness during anaesthesia may occur despite apparently sound anaesthetic management and is usually not associated with pain. However, a few cases may experience excruciating pain and have long term neuropsychiatric sequelae like post-traumatic stress disorder. This adverse event can also have serious medicolegal implications. This article addresses the various contributory factors that may predispose to intra-operative awareness. Preventive measures in the preinduction period as well as intraoperatively are discussed, including the use of depth of anaesthesia monitors. Remedial steps to be taken when such an event occurs are also discussed.

## Introduction

Intraoperative awareness is the unexpected and explicit recall of sensory perception during general anaesthesia. One of the most common concerns of patients about to undergo anaesthesia is that they will remember the intraoperative events. Although the risks associated with anaesthesia have progressively decreased, yet awareness during anaesthesia remains a serious complication with potential long term psychological sequelae.

## Incidence

Awareness during anaesthesia may be experienced by 1 or 2 cases out of every 1000 patients who receive general anaesthesia (0.1-0.2%).^1^,^2^ The overall incidence is higher among obstetric and cardiac cases where it has been quoted at 0.4% and 1.1-1.5% respectively.^3^ In children, the incidence is once again higher at 0.8-1.2%.^4^,^5^

Many patients may not voluntarily report their experiences without being asked directly. Some cases may not recall events shortly after surgery but may recall them 1-2 weeks later.^6^ Intraoperative awareness is therefore best assessed by formally interviewing patients postoperatively. Most of the patients have a vague auditory recall or a sense of dreaming and may not be unduly disturbed by this experience.^7^ In fact dreams may be recalled more often than actual events and occasionally these are very distressing to the patient. In a series of 500 patients anaesthetized with nitrous oxide, Utting reported that 7% patients considered this incidence of dreams to be the worst feature of their experience as against 2% patients who rated the recall of other intra-operative events as the most distressing.^8^ Some patients may even experience severe pain. In a study involving 11,785 patients who underwent general anaesthesia, awareness was reported in 0.18% cases where neuro-muscular blockade was instituted and in 0.1% cases where no muscle paralysis was imposed.^2^ Out of these, 36% patients reported perception of pain ranging from soreness in the throat to pain at the site of incision. Most cases of awareness are inconsequential but some patients experience prolonged and unwanted outcomes like post-traumatic stress disorder and depression.^9^ These late symptoms include nightmares, flashbacks and anxiety and have been reported to occur in upto 33% of the cases who experienced awareness.

This vexing problem of intraoperative awareness was addressed by the Task Force on Intraoperative Awareness which released a ‘Practice Advisory for Intraoperative Awareness and Brain Function Monitoring’ in 2006.^10^ This advisory identified certain patient characteristics and factors that increase the risk of intraoperative awareness and put forth certain recommendations. (Table 1)

**Table 1:** Recommendations of the Practice Advisory for Intraoperative Awareness and Brain Function Monitoring (Apfclbaum JL et al, 2006)^10^

**Preoperative evaluation**
Review patient medical records for risk factors like: –Substance abuse or use –Previous history of intraoperative awareness –History of difficult intubation –Chronic pain patients using high doses of opioids –ASA IV/V –Limited haemodynamic reserve
Interview patient –Obtain history regarding previous experience with anaesthetics
Determine other potential risk factors –Cardiac surgery –Caesarean section –Trauma surgery –Emergency surgery –Decreased anaesthetic doses in the presence of paralysis –Planned use of muscle relaxants during general anaesthesia –Planned use of nitrous oxide- opioid anaesthesia
Patients at high risk should be informed of the possibility of intraoperative awareness when circumstances permit.
**Pre-induction phase of anaesthesia**
Adhere to checklist protocol for checking of anaesthesia machine and equipment Check proper functioning of intravenous access, infusion pumps, connections and backflow valves. Decision to administer benzodiazepines prophylactically should be made on a case to case basis. Intra-operative monitoring Use multiple modalities to monitor depth of anaesthesia – Clinical techniques (e.g. purposeful or reflex movement) – Conventional monitoring systems (e.g. ECG, BP, EtCO_2_ etc.) – Brain function monitoring not routinely indicated for all general anaesthesia cases and should be used for selected patients (e.g. light anaesthesia)
**Postoperative Management**
Interview patient following the adverse event and offer counselling/ psychological support. Initiate occurrence report for quality management.

## Causes

Descriptive studies and case reports suggest that certain patient characteristics may be associated with intraoperative awareness including age, sex, ASA physical status and drug resistance or tolerance. Patients at increased risk for intraoperative awareness include those with a history of substance use or abuse (eg opioids, benzodiazepines, cocaine) and chronic pain patients using high doses of opioids.^1^ A past history of awareness, difficult intubation, ASA physical status of IV/V and a limited haemodynamic reserve are also risk factors.^11^ Procedures which are associated with a higher risk include cardiac surgery, caesarean delivery, trauma and emergency surgery.^12^–^15^ The use of reduced anaesthetic doses in the presence of paralysis, rapid sequence induction and total intravenous anaesthesia have also been implicated.^2^,^16^–^19^

A careful preoperative evaluation is therefore recommended by the Practice Advisory for identifying patients at risk and a thorough review of the patient's medical records, a detailed physical examination and a patient or patient family interview may help identify a vulnerable patient. The Task Force is of the consensus that patients at substantially increased risk of intraoperative awareness should be informed of its possibility by the clinician whenever possible.

The causes of intraoperative awareness are as yet not fully established and may be multifactorial. Four categories of causes have been postulated which are as follows:

- **Unexpected patient specific variability in the dose requirements of anaesthetic drugs**-A certain group of patients have been documented to be more ‘resistant’ to effects of anaesthetics as compared to the others. A younger age group, smoking, long term use of drugs like opiates and alcohol consumption may increase the individual requirement for an anaesthetic drug.^3^ The reason why some patients require a higher dose of anaesthetic is still not very clear. It has been postulated that this variability in dose requirements may be a result of altered gene expression or function of target receptors. In preclinical studies in mice, Cheng and colleagues found that a genetic deficiency in one type of receptor for the inhibitory neurotransmitter, GABA (receptors that contain the α_5_ subunit), conferred resistance to the memory blocking properties of the anaesthetic etomidate.^20^ These receptors are predominantly in the hippocampus region that is critically involved in memory. Other preclinical studies have shown that the expression of this memory blocking receptor changes after long term exposure to alcohol or persistent seizures.^21^,^22^ Concurrent medications can also affect the metabolism and distribution of anaesthetic agents adversely. Polymorphisms for this GABA cc receptor 5 gene (GABRA_5_) exist in the human genome and there are at least 3 distinct messenger RNA isoforms in human adult and foetal brain tissue.^23^ Pharmacogenetics may therefore be an important factor contributing to intraoperative awareness.
- **Requirement for light anaesthesia:** Certain operations like caesarean section may require the anaesthesiologist to aim for lighter anaesthesia. In other cases, patients may often be unable to tolerate a sufficient dose of anaesthetic because of low physiologic reserves related to factors such as poor cardiac function or severe hypovolemia. Judgement about the adequate depth of anaesthesia can thus be imprecise in such patients.
- **Pharmacological masking of signs of inadequate depth of anaesthesia:** Anaesthetic concentrations that block awareness are less than those that prevent motor responses to pain.^24^,^25^ Anonparalyzed but inadequately anaesthetized patient usually communicates by movement. The use of muscle relaxants render such a patient motionless and can lull the anaesthesiologist into a false sense of security. Also the use of drugs like beta blockers or vasodilator agents which have to be given preoperatively for disorders like hypertension may affect intraoperative haemodynamics. Sometimes the anaesthesiologist may use these drugs to tackle intraoperative tachycardia and hypertension without addressing the underlying cause like inadequate depth of anaesthesia. Consequently, physiologic characteristics that would indicate the need for a further deepening of anaesthesia are masked.
- **Machine malfunction or misuse resulting in an inadequate delivery of anaesthesia:** This can be caused by an empty vaporizer, a malfunctioning intravenous pump or a disconnection of its delivery tubing

## Consequences of intraoperative awareness

While pain during surgery is the most distressing feature of awareness, other complaints include the ability to hear conversations during the operation, feelings of anxiety, helplessness, paralysis, panic and impending death.^26^ In some patients awareness causes temporary after effects including sleep disturbances, nightmares and daytime anxiety, which eventually subside. In a small group however, posttraumatic stress disorder develops consisting of repetitive nightmares, irritability and anxiety. Why this disorder develops only in some patients and not in others is not very clear. Factors that are cited include a patient personality, predisposition to mental illness, or the type of emotional response to the disease and surgery.

Intraoperative awareness can thus have long reaching consequences including medicolegal implications. Domino et al, analysed claims from the ASA Closed Claims Project and found that intraoperative awareness accounted for upto 2% of all claims.^11^ What is significant is that this incidence was similar to rates of claims for life threatening complications like myocardial infarction and aspiration pneumonia. Claims were more common in females and where the nitrous oxide-opioid relaxant technique was used.

## Prevention of intraoperative awareness:

Various measures have been recommended to reduce the incidence of intraoperative awareness.

### 1. Preinduction measures:

### i) Premedication with amnesic drugs (e.g. benzo-diazepines):

Prophylactic administration of benzodiazepines as a premedicant especially when light anaesthesia is anticipated, has been advocated. One double blind randomized clinical trial evaluated the efficacy of prophylactic administration of midazolam as an adjuvant during total intravenous anaesthesia and reported a lower frequency of intraoperative awareness in this group as compared to the placebo group.^27^ The Practice Advisory Task Force has however yet not recommended the use of benzodiazepines as a component of anaesthesia to reduce the risk of intraoperative awareness for all patients. Their consensus is that the decision to administer benzodiazepines prophylactically should be made on a case to case basis for selected patients especially those requiring smaller doses of anaesthetics and those undergoing cardiac surgery, emergency surgery, trauma surgery or total intravenous anaesthesia. They have cautioned that delayed emergence may accompany the use of benzodiazepines.^10^

### ii) Meticulous checking of the anaesthesia delivery system before induction:

Cases of intraoperative awareness have been reported to have resulted from anaesthetic concentration delivery errors. Bergman et al, reviewed 8372 incidents reported to the Anaesthetic Incident Monitoring Study and found 81 cases where perioperative recall was consistent with awareness. Awareness was consequent to failure of delivery of volatile anaesthetic in 16 of these patients while in 32 cases a drug error resulting in inadvertent paralysis of an awake patient had occurred.^28^ The Practice Advisory Task Force has strongly recommended that the functioning of anaesthesia delivery systems (eg vaporizers, infusion pumps, fresh gas flows and intravenous lines) should be checked meticulously prior to induction and regular maintenance be carried out.^10^ Regular checking of the anaesthetic in the vaporizer, monitoring of the concentrations of inspired and expired gases and inhalational agents and administration of an anaesthetic infusion via a dedicated intravenous line are simple measures that go a long way in prevention of awareness.

### 2. Intraoperative monitoring:

Intraoperative awareness cannot be measured during the intraoperative period as the recall component of awareness can only be determined postoperatively by obtaining information directly from the patient. The basic question then is whether the use of clinical techniques, conventional monitoring or brain function monitors decreases the occurrence of intraoperative awareness.

### a) Clinical techniques and conventional monitoring:

Clinical techniques used to assess intraoperative consciousness include checking for movement, response to commands, eyelash reflex, pupillary responses, respiratory pattern, perspiration and tearing. Conventional monitoring systems include ECG, blood pressure, heart rate, end tidal anaesthetic analyzer and capnography. No clinical trials or studies have been conducted which specifically examine the sensitivity of these monitoring modalities in detecting intraoperative awareness. Leslie et al., tested the ability of estimated propofol effect- site concentration to predict movement to a stimulus in volunteers during propofol/nitrous oxide anaesthesia.^29^ This was then compared with the predictive abilities of pupillary reflex, systolic blood pressure, BIS and 95% spectral edge frequency of EEG, in the same group. For this comparison, they used the prediction probability (P_K_) which directly compares the performance of indicators having different units of measurement. Numerically, P_K_ is the probability that an indicator predicts correctly which of a pair of randomly selected stimuli, one causing movement and the other not, will result in a movement. An indicator that predicts perfectly whether a movement response will occur has a P_K_ value of 1.0 whereas an indicator that performs no better than chance has a P_K_ value of 0.5. Based on this, their correlational study reported P_K_ values ranging from 0.74 for blood concentration of propofol to 0.86 for BIS. As such, the authors concluded that no significant differences in performance could be demonstrated between these various indicators of anaesthetic depth.

Another study reported significant association between response to command and memory when continuous infusion of propofol was used as the induction anaesthetic.^30^ Wide ranges of mean arterial pressure and heart rate values have been reported during various intraoperative periods and awareness has been found to occur even in the absence of tachycardia or hypertension.^10^

Nevertheless, the Task Force recommends that clinical techniques and conventional monitoring are valuable and should be used to assess intraoperative consciousness. The importance of monitoring the respiration when the patient is not under any neuromuscular paralysis cannot be stressed enough. The Guedel's Stage 3 plane III level of anaesthesia must ideally be achieved before surgery commences so as to ensure adequate anaesthetic depth.

### b)Brain electrical activity monitoring:

Most of the devices designed to monitor brain electrical activity for assessing the anaesthetic effect record EEG activity from electrodes placed on the forehead. Systems can be further divided into those that process spontaneous EEG and electromyographic activity and those that acquire evoked responses to auditory stimuli.

### I. Spontaneous electroencephalographic activity monitors:

### (1) Bispectral index: (Aspect Medical systems, MA)

The BIS converts a single channel of frontal EEG into an index of hypnotic level. To compute the BIS, several variables derived from the EEG time domain and frequency domain are combined into a single index of hypnotic level. Targeting a range of BIS values 40-60 is advocated to prevent awareness during anaesthesia while allowing a reduction in the administration of anaesthetic agents.^31^

Several randomized controlled studies have compared outcomes with BIS guided anaesthetic administration versus standard clinical practice without BIS. In the B-Aware study that included 2500 patients at high risk of intraoperative awareness, explicit recall occurred in 0.17% of patients when BIS monitors were used as compared to 0.91% in patients treated by routine clinical practice.^7^

Another nonrandomized comparison of the use of BIS monitoring versus a cohort of historic controls in a group of 12,771 patients found explicit recall occurring in 0.04% of BIS monitored patients versus 0.18% of the historic controls.^32^ Other studies conducted to determine BIS values associated with intraoperative awareness reported no statistically significant difference when BIS was used (0.18%) as compared to when BIS was not used (0.1%).^1^

A more recent study was conducted to determine whether a BIS protocol was better than a protocol based on a measurement of end tidal anaesthetic gas(ETAG) for decreasing anaesthetic awareness in high risk patients. 2000 patients were randomly assigned to BIS-guided anaesthesia (target BIS range of 40-60) or ETAG-guided anaesthesia (target ETAG range of 0.7 to 1.3 MAC). The patients were assessed for awareness postoperatively at 0-24 hrs, 24-72 hrs and at 30 days following surgery. It was found that intraoperative awareness occurred even when BIS values and ETAG concentrations were within target ranges and the authors did not support the routine use of BIS monitors as a part of a standard practice during general anaesthesia.^33^ Several intraoperative events unrelated to titration of anaesthetic agents can produce rapid changes in BIS values(eg cerebral ischaemia, hypoperfusion, gas embolism, unrecognized haemorrhage, inadvertent blockage of anaesthetic drug delivery).^34^–^37^ There are other case reports that suggest that routine intraoperative procedures (eg. activation of electromagnetic devices, patient warming or cooling) may interfere with BIS functioning.^38^–^41^ Mychaskiv et al, demonstrated the failure of BIS as a reliable monitor of the depth of anaesthesia.^42^ Their patient had the horrifying experience of both hearing the sternal saw as well as feeling his chest being cut open during cardiac surgery. This recall of intraoperative events occurred at a BIS of 47 with nitrous oxide and sevoflurane anaesthesia. Other authors have also reported incidences of intraoperative awareness despite monitored values of BIS indicating an adequate depth of anaesthesia.^43^

### (2) Entropy: (GE Healthcare Technologies Waukesha, WI)

Entropy describes the irregularity, complexity or unpredictability characteristics of a signal. A single sine wave represents a completely predictable signal (entropy=0) whereas noise from a random number generator represents entropy =1. State entropy (SE) is an index ranging from 0-91 (awake) computed over the frequency range from 0.8 to 32 Hz reflecting the cortical state of the patient. Response entropy (RE) is an index ranging from 0-100 (awake) computed over a frequency range from 0.8-47 Hz containing the higher electromyographic dominated frequencies and will therefore respond to increased electromyographic activity resulting from inadequate analgesia. Correlational studies report the following P_k_ values for loss of consciousness: for RE, 0.83-0.97; for SE, 0.81-0.90. No clinical trials however are available that conclusively show that entropy monitoring reduces the incidence of intraoperative awareness.

### (3) Narcotrend (Monitor Technik, Germany)

The Narcotrend is derived from a system developed for the visual classification of the EEG patterns associated with various stages of sleep. After artefact exclusion and Fourier transformation, the original electronic algorithm classified the frontal EEG according to: A (awake), B (sedated), C (light anaesthesia), D (general anaesthesia), E(general anaesthesia with deep hypnosis), F (general anaesthesia with increasing burst suppression). In a recent iteration of the software, the alphabetical scale has been translated into a dimensionless index scaled from 0 (deeply anaesthetized) to 100 (awake). Reported mean Narcotrend values are as follows: after induction-72-80 and at emergence-80.^44^

There are few studies on the reliability of Narcotrend as an aid to reduce awareness. Russel used the ‘isolated forearm technique’ to check for the presence of intraoperative consciousness during general anaesthesia.^45^ This study concluded that the Narcotrend was unable to differentiate reliably between conscious and unconscious patients during general anaesthesia when neuromuscular blocking agents were used.

### (4) Patient State Analyser (Physiometrix, North Billerica, MA)

The patient state index (PSI) is derived from a four channel electroencephalograph. The derivative of the Patient State Index is based on the observation that there are reversible spatial changes in power distribution of quantitative EEG at loss and return of consciousness. The PSI has a range of 0-100 with decreasing values indicating lower levels of consciousness and sedation. The reliability of PSI is however debatable. Sneider et al, studied the ability of PSI and BIS to detect awareness in 40 patients subjected to anaesthesia with sevoflurane-remifentanil/propofol-remifentanil combination. They concluded that despite significant differences between mean values at responsiveness and nonresponsiveness for BIS and PSI, neither measure was sufficient to detect awareness in an individual patient.^46^ A subsequent study has however shown PSI to be a useful indicator of the level of hypnosis under general anaesthesia.^47^

### (5) SNAP index (Everest Biomedical Instruments, Chesterfield, MO)

The SNAP II calculates a “SNAP index” from a single channel of EEG. The index calculation is based on a spectral analysis of EEG activity in the 0-18 Hz and 80-420 frequency ranges and a burst suppression algorithm. There are no published data on the actual algorithm used to calculate the SNAP index, which is based on a composite of both low frequency (0-40 Hz) and high frequency (80-420 Hz) component.

### (6) Cerebral State Monitor (Danmeter A/S, Odense, Denmark)

This is a hand held device that analyses a single channel EEG and presents a Cerebral State “Index” scaled from 0-100. It also provides EEG suppression percentage and a measure of electromyographic activity (75-85 Hz). No literature is available that has examined the impact of these two monitors on the incidence of intraoperative awareness.

## II. Evoked brain electrical activity monitors:

### Auditory Evoked Potential Monitor (Danmeter)

Auditory evoked potentials are the electrical responses of the brain stem, the auditory radiation and the auditory cortex to auditory sound stimuli in the form of clicks delivered via headphones. The brainstem response is relatively insensitive to anaesthetics whereas early cortical responses called midlatency auditory evoked potentials (MLAEPs) change in a predictable manner with increasing concentrations of volatile and intravenous anaesthetics. Increasing anaesthetic concentrations lead to an increased latency and reduced amplitude of the various waveform components. From a mathematical analysis of the AEP waveform, the device generates a AEP index (AAI) that provides a correlate of anaesthetic concentration. This AEP index is scaled from 0-100 and the AAI corresponding with a low probability of consciousness is <25. Randomized controlled trials comparing MLAEP monitoring to standard clinical anaesthesia practices without MLAEPs reported reduced times to eye opening or orientation.^48^ Another study reported a P_k_ value of 0.99 for awareness after LMA insertion.^49^

## Present status of brain function monitors

The use of a brain function monitor is dedicated to the assessment of effects of anaesthetics on the brain and correlation with the depth of anaesthesia. Although we are familiar with the effects of anaesthetics, our knowledge of their exact site and mechanism of action still remains limited. Surface electrodes for cortical EEG measurements are unlikely to reveal drug action at the level of critical memory centres like the hippocampus. Also, the measured values by these monitors do not have a uniform sensitivity across different anaesthetic drugs and types of patients. Thus it is possible that patients can have awareness despite apparently low BIS values. The incidence of awareness depends on the type of surgery, the anaesthetics used and the timing of and technique of evaluating awareness and recall.^50^ Moreover, artefacts can be introduced by the use of cautery, lasers, patient warmers etc. As such the clinical applicability of these monitors in the prevention of intraoperative awareness has not been established. Although isolated reports of a decrease in the frequency of awareness in high risk cases are available, there is still insufficient evidence to justify a standard guideline for the use of these monitors to reduce the risk of awareness during general anaesthesia. Another important consideration is the extra financial burden imposed by the use of these monitors. Routine awareness monitoring with a proprietary device in most patients undergoing anaesthesia would add about £30 million to UK healthcare costs.^51^ Although no figures are available for India, the economic aspect may be an important consideration. Hence, inspite of a large variety of brain function monitors being available, the consultants participating in the Practice Advisory for Intraoperative Awareness do not support the use of a brain electrical activity monitor to decrease the risk of intraoperative awareness for all patients. They recommend its use for patients with conditions that place them at higher risk such as trauma surgery, caesarean, total intravenous anaesthesia and patients requiring smaller doses of general anaesthetics. The Advisory recommends that intraoperative monitoring of the depth of anaesthesia for the purpose of minimizing the occurrence of awareness should rely on multiple modalities including clinical techniques (e.g. reflex movement) and conventional monitoring systems (e.g. ECG, blood pressure, end tidal anaesthetic analyzer and capnography). Ensuring adequate delivery of anaesthetics assumes even more importance when neuromuscular blocking drugs are used.

### Recommendations for management of post anaesthesia awareness

Measures that have been recommended should this adverse event occur include the following:

1. Providing a postoperative structured interview and a questionnaire to the patient so as to define the nature of the intraoperative awareness episode, after it has been reported.
2. Offering postoperative counselling or psychological support.

No studies are available that demonstrate an improvement in the patient's well being following the use of questionnaires or interviews when intraoperative awareness has occurred. However, the Task Force does recommend that a detailed account of the patient's experience be obtained. He or she should be reassured and some explanation for what has happened and why should be given (eg. the necessity to administer light anaesthesia due to cardiovascular instability). Details of the incident should be placed in the patient's records so as to guide the anaesthesiologist for management of future anaesthetics. The patient should be offered psychological or psychiatric support. The details of the interview should be recorded in the patients chart and the surgeon, patient's nurse, hospital lawyer and physician's insurer should be notified. During the hospital stay, the patient should be visited daily to look for psychological sequelae like sleep disturbances, day time anxiety etc. Following discharge, contact by telephone should be maintained till the patient is fully recovered. Early referral to the psychiatrist or psychologist should be done whenever necessary so as to reduce the incidence of post traumatic stress disorder. Finally an occurrence report regarding the event should be completed for the purpose of quality management.

Intraoperative awareness is an alarming complication both for the patient and anaesthesiologist alike. Although the incidence is rare, yet it can have considerable potential for severe emotional distress in the patient as well as professional, personal and financial consequences for the anaesthesiologist. It may be caused by a poor technique or equipment malfunction but its occurrence does not necessarily indicate these problems. While a few simple measures may go a long way in reducing its incidence, further research needs to be directed towards eliminating this unexpected anaesthetic problem. Finally, a sympathetic approach and good psychological support will help reduce the patient's trauma following this adverse event.
